# Radiomics analysis combining gray-scale ultrasound and mammography for differentiating breast adenosis from invasive ductal carcinoma

**DOI:** 10.3389/fonc.2024.1390342

**Published:** 2024-07-09

**Authors:** Wen Li, Ying Song, Xusheng Qian, Le Zhou, Huihui Zhu, Long Shen, Yakang Dai, Fenglin Dong, Yonggang Li

**Affiliations:** ^1^ Department of Radiology, The First Affiliated Hospital of Soochow University, Suzhou, Jiangsu, China; ^2^ Department of Ultrasound, Huadong Sanatorium, Wuxi, Jiangsu, China; ^3^ Department of Ultrasound, The First Affiliated Hospital of Soochow University, Suzhou, Jiangsu, China; ^4^ Suzhou Institute of Biomedical Engineering and Technology, Chinese Academy of Sciences, Suzhou, Jiangsu, China; ^5^ School of Biomedical Engineering (Suzhou), Division of Life Sciences and Medicine, University of Science and Technology of China, Hefei, Anhui, China; ^6^ Department of Radiology, Suzhou Xiangcheng District Second People’s Hospital, Suzhou, Jiangsu, China; ^7^ Institute of Medical Imaging, Soochow University, Suzhou, Jiangsu, China; ^8^ National Clinical Research Center for Hematologic Diseases, The First Affiliated Hospital of Soochow University, Suzhou, Jiangsu, China

**Keywords:** invasive ductal carcinoma, breast adenosis, radiomics, gray-scale ultrasound, mammography

## Abstract

**Objectives:**

To explore the utility of gray-scale ultrasound (GSUS) and mammography (MG) for radiomic analysis in distinguishing between breast adenosis and invasive ductal carcinoma (IDC).

**Methods:**

Data from 147 female patients with pathologically confirmed breast lesions (breast adenosis: 61 patients; IDC: 86 patients) between January 2018 and December 2022 were retrospectively collected. A training cohort of 113 patients (breast adenosis: 50 patients; IDC: 63 patients) diagnosed from January 2018 to December 2021 and a time-independent test cohort of 34 patients (breast adenosis: 11 patients; IDC: 23 patients) diagnosed from January 2022 to December 2022 were included. Radiomic features of lesions were extracted from MG and GSUS images. The least absolute shrinkage and selection operator (LASSO) regression was applied to select the most discriminant features, followed by logistic regression (LR) to construct clinical and radiomic models, as well as a combined model merging radiomic and clinical features. Model performance was assessed using receiver operating characteristic (ROC) analysis.

**Results:**

In the training cohort, the area under the curve (AUC) for radiomic models based on MG features, GSUS features, and their combination were 0.974, 0.936, and 0.991, respectively. In the test cohort, the AUCs were 0.885, 0.876, and 0.949, respectively. The combined model, incorporating clinical and all radiomic features, and the MG plus GSUS radiomics model were found to exhibit significantly higher AUCs than the clinical model in both the training cohort and test cohort (*p*<0.05). No significant differences were observed between the combined model and the MG plus GSUS radiomics model in the training cohort and test cohort (*p*>0.05).

**Conclusion:**

The effectiveness of radiomic features derived from GSUS and MG in distinguishing between breast adenosis and IDC is demonstrated. Superior discriminatory efficacy is shown by the combined model, integrating both modalities.

## Introduction

Breast adenosis, a prevalent proliferative breast condition lacking atypia (1), has an uncertain etiology. However, Endocrine alterations and disorders, such as increased levels of estrogen, a shortage in progesterone, hyperprolactinemia, imbalances in thyroid hormones, stress, and deficiencies in unsaturated fatty acids, may contribute to its development (2). Pathologically, breast adenosis manifests as an enlargement of the lobule and terminal ductal lobular unit, characterized by an increased number of ductules and acini within the lobule (3). Radiologically, some instances of breast adenosis may be erroneously interpreted as invasive ductal carcinoma (IDC). While breast adenosis typically only requires observation and monitoring, IDC necessitates a comprehensive treatment plan involving surgery, radiation, and chemotherapy (4, 5). Therefore, accurate preoperative differentiation between breast adenosis and IDC is imperative for precise diagnosis and optimal patient management.

Gray-scale ultrasound (GSUS) and mammography (MG) are widespread screening tools for breast cancer globally, providing crucial diagnostic guidance (6, 7). However, Ozturk et al. (8). demonstrated the inherent difficulty in distinguishing breast adenosis from IDC using MG and ultrasound (US) due to the absence of characteristic features. Additionally, MRI is expensive and time-consuming for routine examination in breast adenosis diagnosis, while CT carries a higher radiation dose risk and offers lower resolution in differentiating the fine structures of breast tissue (9–11).

Radiomics is a data mining approach aimed at extracting high-dimensional data from clinical images to build diagnostic and predictive models for addressing relevant clinical questions (12, 13). Radiomics has long been widely employed in MG and US images to enhance the efficiency and accuracy of breast cancer screening (14, 15). Nevertheless, the effectiveness of radiomics based on single-modality medical images is limited, as these images capture only a fraction of tumor information due to their imaging principles (16). There is growing interest in using multimodal radiomics to acquire a more complete and nuanced understanding of tumor properties, such as shape, size, and texture (16, 17). However, limited studies have investigated the use of radiomics to differentiate between breast adenosis and IDC, particularly using the combination of GSUS and MG. Therefore, this study aims to develop models using radiomic features extracted from GSUS and MG images, along with clinical data, to differentiate between breast adenosis and IDC.

## Materials and methods

### Subjects

**Figure 1:**
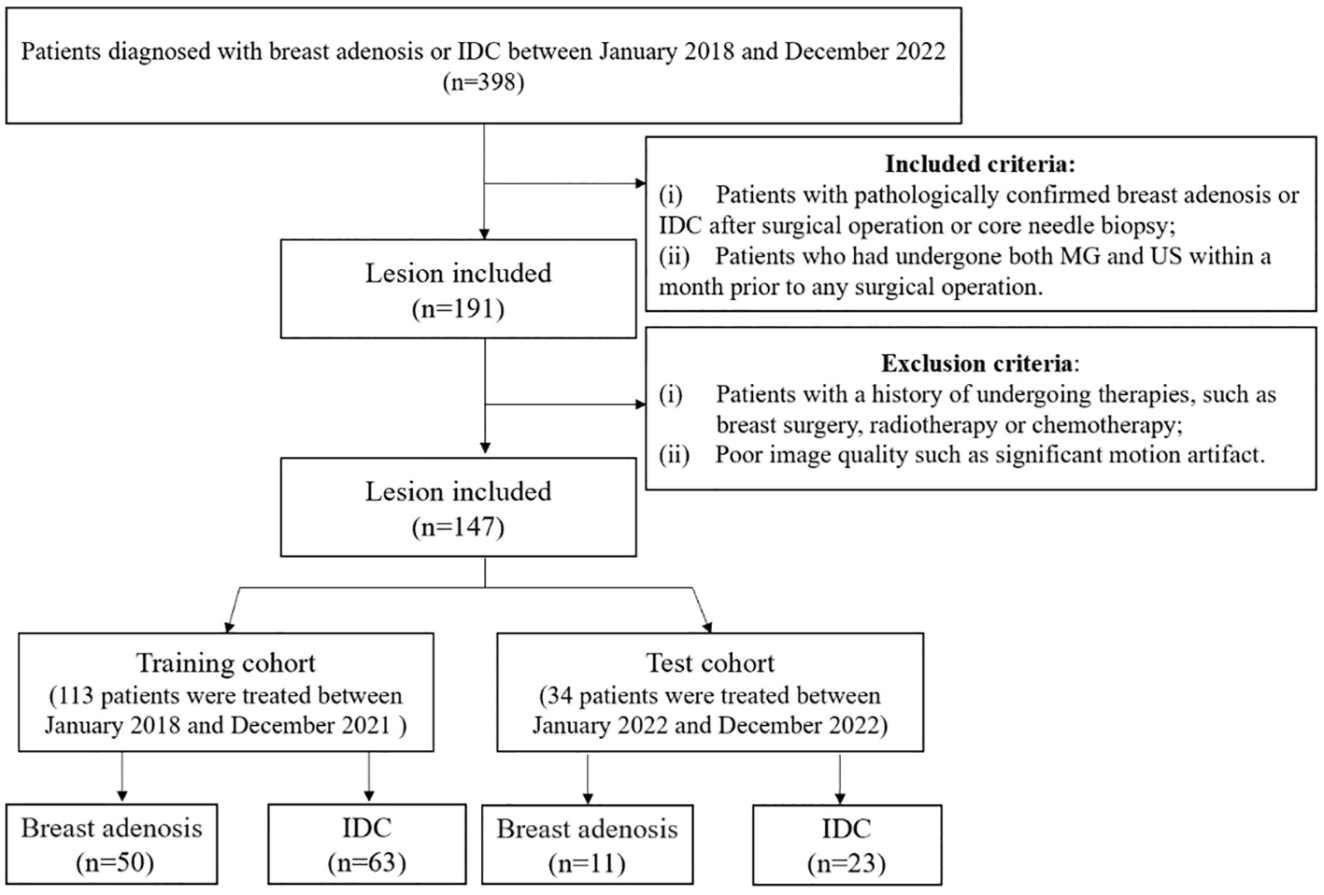
The flowchart illustrates the inclusion and exclusion criteria for study subjects. MG, mammography; US, ultrasound; IDC, invasive ductal carcinoma.

This retrospective study received approval from our institution’s independent ethics committee, and the need for informed consent was waived. Patients with histologically confirmed breast adenosis or invasive ductal carcinoma (IDC) enrolled between January 2018 and December 2022. Inclusion criteria were: (i) patients with pathologically confirmed breast adenosis or IDC post-surgical operation or core needle biopsy; (ii) patients who had undergone both mammography (MG) and ultrasound (US) within a month prior to any surgical operation. Exclusion criteria were: (i) patients with a history of undergoing therapies such as breast surgery, radiotherapy, or chemotherapy; (ii) poor image quality, including significant motion artifact. A total of 147 patients, 61 with breast adenosis (mean age, 45 ± 12 years; range, 26-69 years) and 86 with IDC (mean age, 52 ± 12 years; range, 31-74 years), were included. All patients had a single and unilateral lesion. Based on the time sequence of patients receiving treatment, they were divided into training and test cohorts. The training cohort comprised 113 patients treated between January 2018 and December 2021, while the test cohort comprised 34 patients treated between January 2022 and December 2022. Clinical data, including age, family history of breast cancer, and menopausal status, were collected from medical records. **Figure 1** illustrates the patient selection process.

### Imaging acquisition and interpretation

All patients underwent a pre-surgical US examination, positioned supine with hands raised above their heads for full breast exposure. Color Doppler ultrasound instruments used included GE LOGIQ E9 (General Electric Company, Boston, USA), MyLab™ ClassC (Esaote, Genoa, Italy), TOSHIBA APLIO 500 (Toshiba, Tokyo, Japan), or HITACHI ARIEETTA 70 (HITACHI Ltd, Tokyo, Japan) with a linear array probe and a frequency of 9-12 MHz. The examinations were conducted by ultrasound practitioners with 10 years of experience in breast ultrasonography, trained in standardized chart storage. The field of view was adjusted to encompass the pectoralis muscle at its most profound point within the photograph. The focus was situated slightly beneath the lesion. The standard stored image of a breast lesion comprised of a minimum of two vertical slices, with one of them displaying the lesion’s maximum diameter. The image exhibiting the clearest and most comprehensive presentation of the lesion was chosen. A digital mammography machine (Hologic Selenia, Hologic Medical Systems, Boston, USA) was employed to capture images in mediolateral oblique (MLO) and cranial-caudal (CC) positions.

### Image analysis

Two radiologists with 10 and 15 years of MG diagnostic experience, respectively, and another two radiologists with 9 and 12 years of breast US diagnostic experience, respectively, independently assessed the images without access to clinical and pathological information. They recorded imaging features of lesions on MG and US, including glandular type, architectural distortion, microcalcification morphology, mass, asymmetric focal density, shape, orientation, posterior feature, margin, calcification, vascularity grade, and echo pattern. In case of disagreement, a final consensus was achieved through discussion. Lesions were categorized based on the 5th edition of the Breast Imaging Reporting and Data System (BI-RADS) (18).

### Tumor segmentation and radiomics feature extraction

Breast lesions in the MLO and CC positions of MG images were manually segmented by radiologist 1 (with 5 years of MG diagnostic experience), confirmed by radiologist 2 (with 10 years of experience), and adjusted if necessary. For GSUS images, breast lesions were manually segmented by radiologist 3 (with 7 years of experience in breast GSUS diagnosis), confirmed by radiologist 4 (with 15 years of experience), and adjusted if necessary. Radiologists were aware of the lesion locations but remained blinded to the clinical and pathological information of the patients.

**Figure 2:**
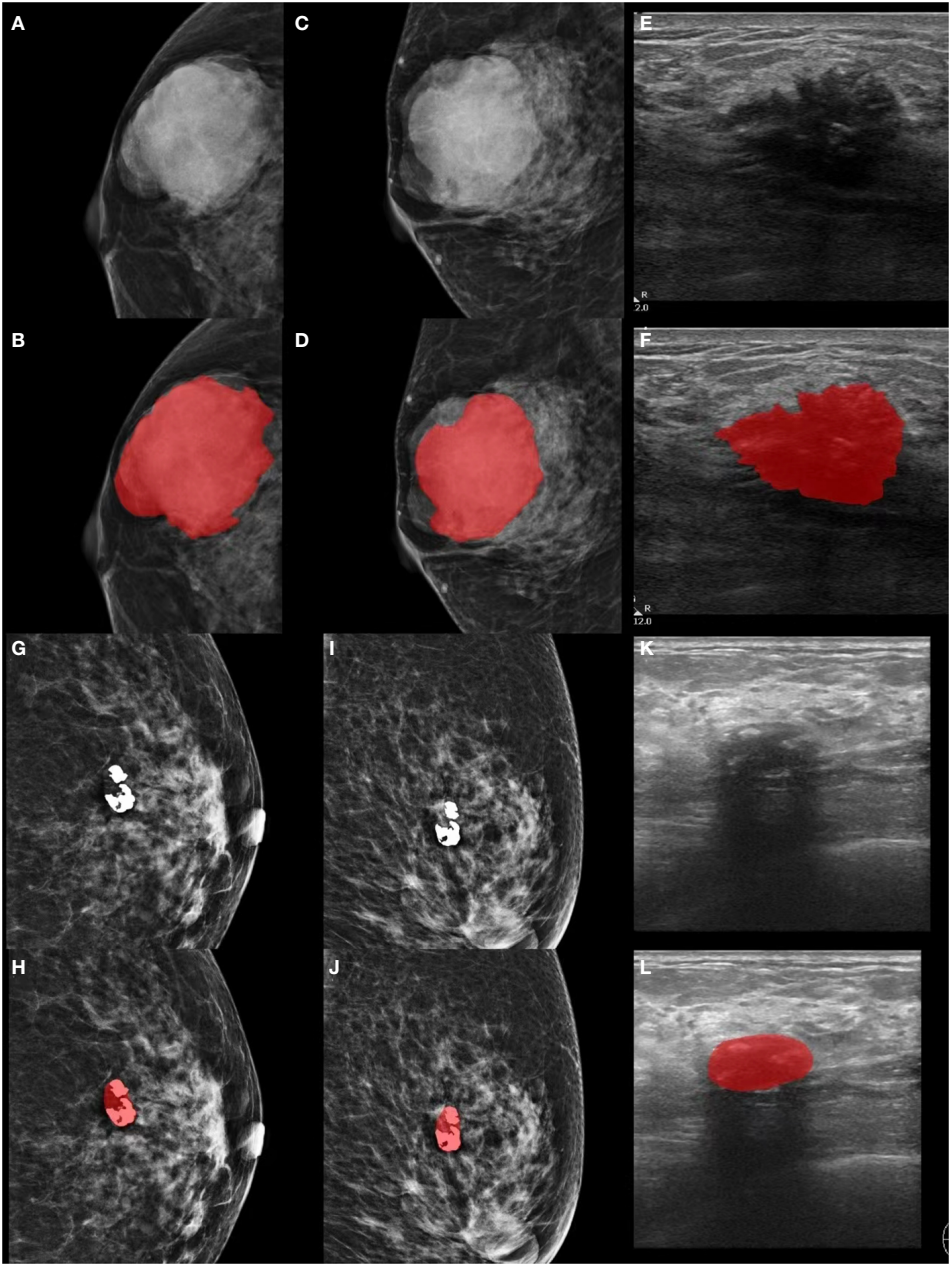
Lesion segmentation in MG and GSUS images. **(A-F)** Displaying the original MG, GSUS images, and segmentation of a 32-year-old female patient with confirmed breast adenosis. **(G-L)** Presenting the original MG, GSUS images, and segmentation of a 57-year-old female patient with confirmed IDC. MG, mammography; GSUS, gray-scale ultrasound; IDC, invasive ductal carcinoma.

Manual segmentation utilized the open-source ITK-SNAP software (version 3.8.0, http://www.itksnap.org). In cases of uncertain lesion boundaries, a final consensus was achieved through discussion, as illustrated in **Figure 2**.

The radiomics features of the GSUS and MG images were extracted using an artificial intelligence-assisted diagnosis modeling software based on Pyradiomics (version 2.2.0) (19). The extracted features were categorized into seven groups: shape features, first-order statistical features, gray-level co-occurrence matrix (GLCM) features, gray-level run length matrix (GLRLM) features, gray-level size zone matrix (GLSZM) features, neighboring gray-tone difference matrix (NGTDM), and gray-level dependence matrix (GLDM) features. Radiomic features were generated on both the original and pre-processed images using seven filters, including Laplacian of Gaussian (LoG) (σ= 0.5, 1.0, 1.5), Logarithm, Square, Square root, Exponential, Wavelet, and Gradient.

The segmentation process was repeated in 30 randomly selected patients by radiologist 1 and radiologist 3 after 2 months. Intraclass correlation coefficients (ICCs) were calculated to assess the intra-observer reproducibility of radiomic features. An ICC ≥ 0.8 indicates high agreement, 0.5 to 0.79 indicates moderate agreement, and < 0.5 indicates low agreement (20, 21).

### Feature selection, model construction and validation

**Figure 3:**
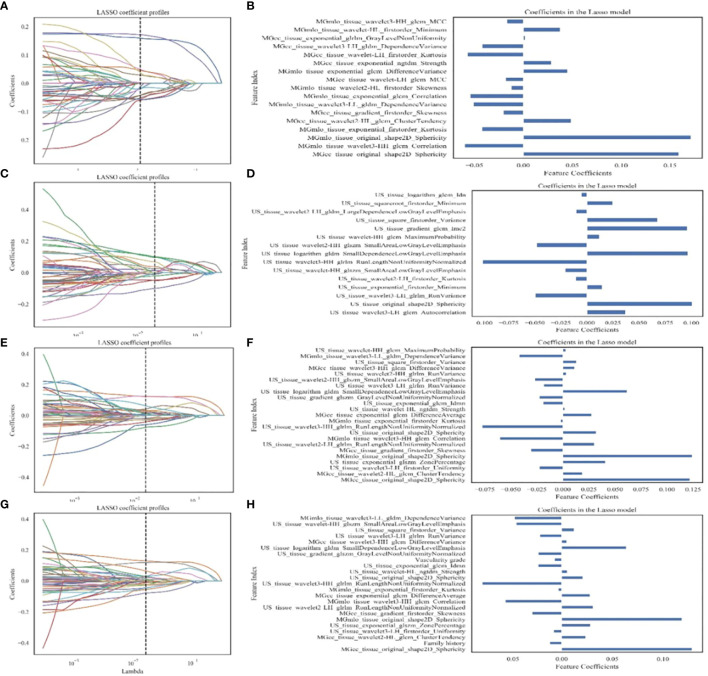
Feature extraction and filtering. **(A, C, E, G)** Depicting LASSO coefficient profiles (y-axis) for radiomics features from MG images, GSUS images, combined radiomics features of MG and GSUS images, and clinical features combined with MG and GSUS radiomics features. **(B, D, F, H)** Illustrating selected features and coefficients in the models. MG, mammography; GSUS, gray-scale ultrasound; IDC, invasive ductal carcinoma.

All radiomic features were normalized to a zero mean and unit variance using Z-score. A comprehensive feature selection process was conducted as follows. Firstly, features with low variance (<0.1) were eliminated. Secondly, Welch’s t-test compared the remaining features between breast adenosis and IDC groups, with a *p*-value < 0.05 considered statistically significant, and insignificant features were removed. Thirdly, We retained a relatively large number of the top 50 features in the minimum redundancy maximum relevance (mRMR) method, in order to reduce the redundancy between features and retain relevant features, while reducing the possibility of mRMR method deleting features helpful for the identification of breast adenosis and IDC. Finally, the least absolute shrinkage and selection operator (LASSO) regression determined the most discriminant feature subset (**Figures 3A, C, E, G**). The optimal penalization parameter λ value, minimizing the mean square error (MSE), was automatically chosen for LASSO using an estimator with built-in cross-validation capability. Subsequently, the L2-regularized logistic regression (LR) model (22, 23) was trained based on the selected clinical, GSUS, and MG image features, along with radiomic features. The GridSearchCV was employed from sklearn package to carry out a grid search to find the optimal value of the L2 regularization parameter by using fivefold cross-validation, repeated five times on the training cohort (24). Separate models were established for the clinical model, MG-based radiomics model (Rad-MG model), GSUS-based radiomics model (Rad-GSUS model), MG plus GSUS radiomics model (Rad-MG-GSUS model), and combined model integrating clinical features, MG, and GSUS radiomics features. The discriminatory ability of each model was assessed using the receiver operating characteristic (ROC) curve and decision curve analysis (DCA). The area under the ROC curve (AUC), sensitivity, specificity, and accuracy were calculated. Model comparisons within the training and test cohorts were based on Delong test results.

### Statistical analysis

Statistical analyses were conducted using SPSS 25.0 (IBM Corp., Armonk, NY, USA) and Python 3.6 (Python Software Foundation, Beaverton, OR, USA) software. The Kolmogorov-Smirnov test assessed the normality of quantitative data. Quantitative data conforming to a normal distribution were presented as mean ± standard deviation; otherwise, data were expressed as the median (interquartile range). Qualitative data were reported as numbers. The comparison of quantitative data utilized the independent sample t-test or Mann-Whitney U-test, while the x^2^ test was employed for qualitative data comparison. LASSO logistic regression, ROC curves, and LR were implemented using the sklearn package. Model performance was evaluated through the Delong test and decision curve analysis (DCA). A two-tailed *p*-value < 0.05 was considered indicative of statistical significance.

## Results

### Patient profiles

**Figure 4:**
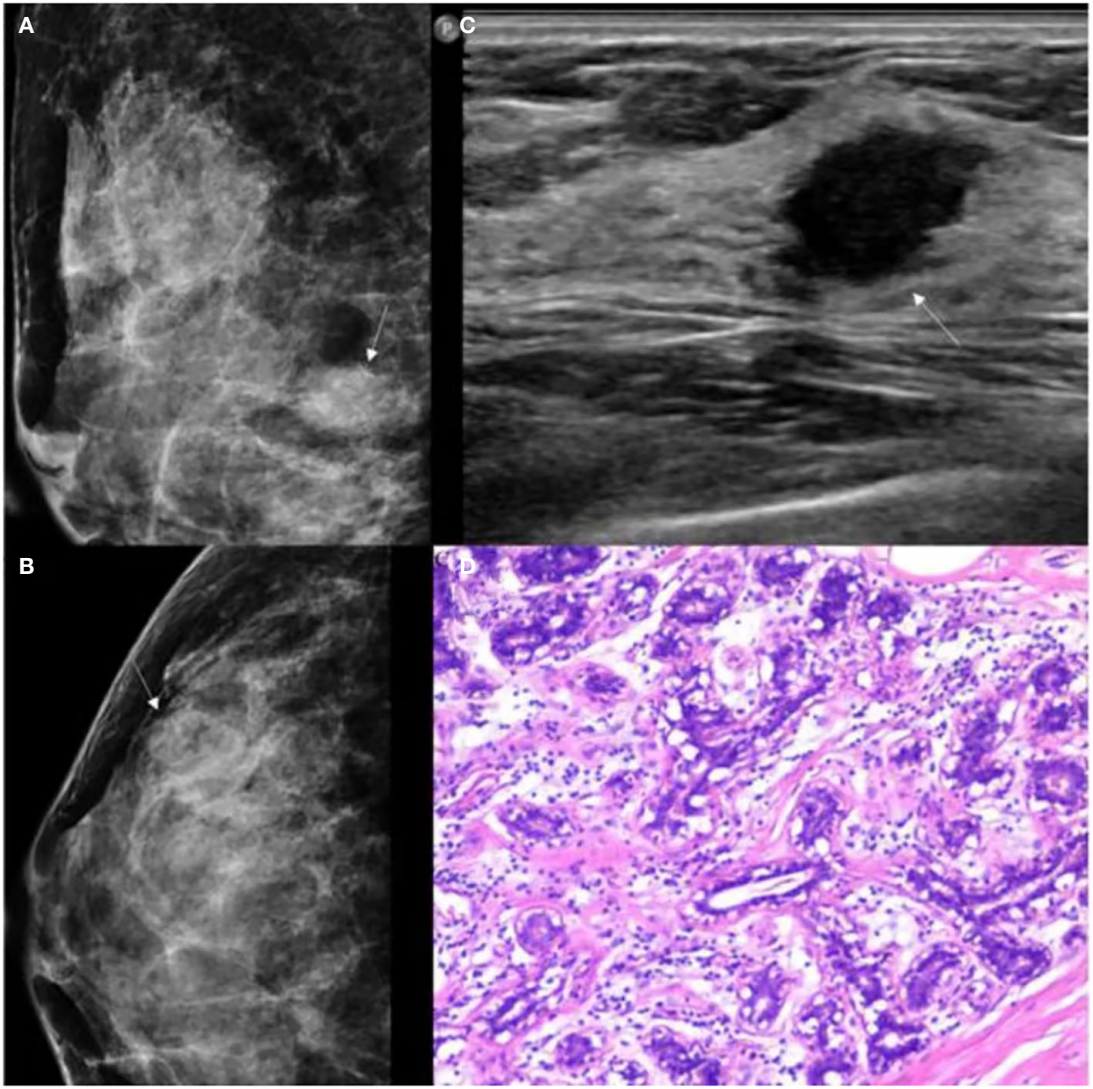
GSUS, MG and histopathologic findings of a 37-year-old woman with breast adenosis. **(A, B)** The MG presents an oval shape, ill-circumscribed isodense mass (white arrow); **(C)** The GSUS presents a irregular shape, ill-circumscribed homogeneity mass (white arrow); **(D)** The sample (hematoxylin and eosin) with breast adenosis (100x). MG, mammography; GSUS, gray-scale ultrasound.

**Figure 5:**
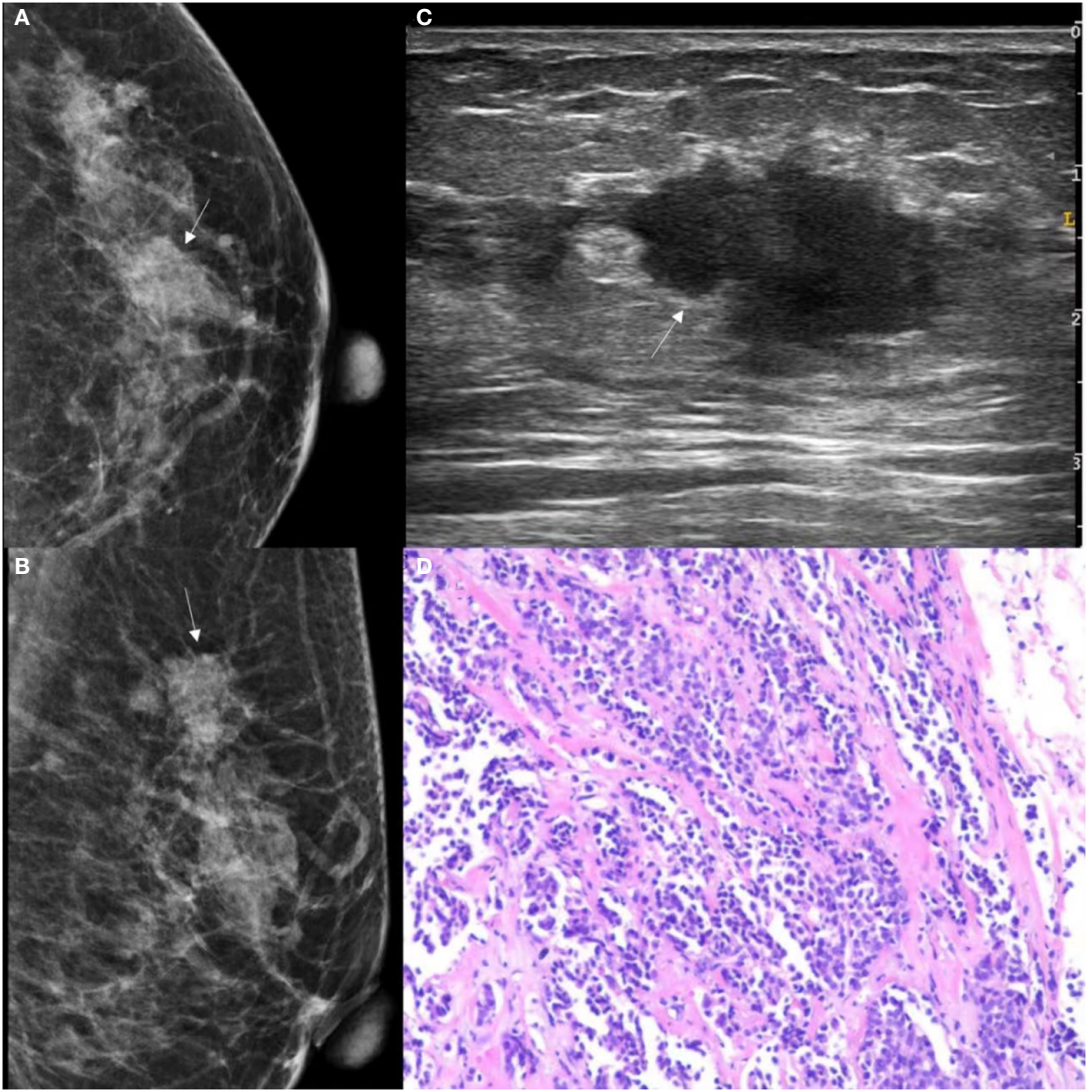
GSUS, MG and histopathologic findings of a 48-year-old woman with IDC. **(A, B)** The MG presents an irregular shape, ill-circumscribed isodense mass (white arrow) with a microlobulated margin; **(C)** The GSUS presents an irregular shape, ill-circumscribed heterogeneity mass (white arrow); **(D)** The sample (hematoxylin and eosin) with IDC (100x). MG, mammography; GSUS, gray-scale ultrasound; IDC, invasive ductal carcinoma.

A total of 147 patients were included in the study, comprising 61 with breast adenosis and 86 with IDC. The training cohort comprised 113 patients (breast adenosis: 50; IDC: 63), while the test cohort included 34 patients (breast adenosis: 11; IDC: 23). Histopathological examination results showed 8 cases of pure adenosis and 53 cases of adenosis mixed with fibrocystic changes among the adenosis lesions. **Table 1** presents the clinical, US, and MG imaging features. Significant differences in age, menopausal status, family history, microcalcification morphology, and vascularity grade were observed between the breast adenosis and IDC groups in the training cohort (*p*<0.05). No significant difference was found between the two groups in glandular type, architectural distortion, mass, asymmetric focal density, shape, orientation, posterior feature, margin, calcification, and echo pattern (*p*>0.05). The GSUS features, MG features and histopathological characteristics of breast lesions or IDC are summarized in **Figures 4** and **5**.

**Table 1:** Demographic characteristics and imaging features of GSUS and MG.

	Training cohort (n=113)			Validation cohort (n=34)		
	breast adenosis (n=50)	IDC (n=63)	*P*	breast adenosis (n=11)	IDC (n=23)	*P*
Age	45 + 12	52 ± 12	0.002^*^	48 ± 12	55 ± 11	0.138
Menopausal status			0.021^*^			0.434
postmenopausal	20	39		6	17	
premenopausal	30	24		5	6	
Family history			0.042^*^			0.324
no	49	55		10	23	
yes	1	8		1	0	
Mammography features						
Glandular type			0.155			1.000
entirely fatty	1	1		0	0	
scattered fibroglandular	1	8		2	4	
heterogeneously dense	42	49		9	19	
extremely dense	6	5		0	0	
Architectural distortion			0.066			1.000
no	47	52		11	22	
yes	3	11		0	1	
Microcalcification morphology			0.013^*^			0.615
no	30	23		5	10	
punctate	12	24	<0.001^*^	4	10	0.442
amorphous	1	3		0	0	
coarse heterogeneous	4	1		1	0	
fine pleomorphic	1	1		0	1	
fine linear/fine-linear branching	2	11		1	2	
Mass			0.574			0.064
no	16	21		6	6	
circumscribed	6	4	0.328	1	0	0.227
ill-circumscribed	28	38		4	17	
Asymmetric focal density			0.292			1.000
no	40	55		9	20	
yes	10	8		2	3	
Ultrasound features						
Shape			0.699			0.300
regular	11	12		3	2	
irregular	39	51		8	21	
Orientation			0.181			0.535
orientation	45	51		11	20	
not parallel	5	12		0	3	
Posterior feature			0.469			1.000
no posterior feature	44	58		11	22	
shadowing	6	5		0	1	
Margin			0.279			0.070
circumscribed	12	10		4	2	
not circumscribed	38	53		7	21	
Calcification			0.653			0.053
no	33	39		10	12	
In a mass	17	24		1	11	
Vascularity grade			<0.001^*^			0.152
grade 0~I	38	26		8	10	
grade II~III	12	37		3	13	
Echo pattern			0.568			1.000
homogeneity	10	10		1	1	
heterogeneity	40	53		10	22	

Data are shown as mean ± standard deviation, or n. *P value < 0.05, with statistical difference.

IDC, invasive ductal carcinoma; MG, mammography; GSUS, gray-scale ultrasound.

### Feature extraction and selection

A total of 4491 radiomics features were extracted from GSUS (1497 features) and MG (2994 features) images for each patient. The intraobserver ICC ranged from 0.76 to 0.97, indicating a good reproducibility of radiomics feature extraction. 109 features with an ICCs value less than 0.80 were excluded. Univariate analysis of five features (age, family history of breast cancer, and menopausal status, microcalcification morphology, vascularity grade) with statistical differences in the training cohort was used to construct the clinical model. Radiomics features of four radiomics signatures were shown in **Table 2**, **Figures 3B, D, F, H**.

**Table 2:** Radiomics features of four models.

Rad-MG model	Rad-GSUS model	Rad-MG-GSUS model	Combined model
MGcc_tissue_original_shape2D_Sphericity	US_tissue_wavelet3-LH_glcm_Autocorrelation	MGcc_tissue_original_shape2D_ Sphericity	MGcc_tissue_original_shape2D_ Sphericity
MGmlo_tissue_wavelet3- HH_glcm_Correlation	US_tissue_original_shape2D_Sphericity	MGcc_tissue_wavelet2-HL_glcm_ClusterTendency	Family history
MGmlo_tissue_original_ shape2D_Sphericity	US_tissue_wavelet3-LH_glrlm_RunVariance	US_tissue_wavelet3-LH_firstorder_Uniformity	MGcc_tissue_wavelet2-HL_glcm_ ClusterTendency
MGmlo_tissue_exponential_ firstorder_Kurtosis	US_tissue_exponential_firstorder_Minimum	US_tissue_exponential_glszm_ZonePercentage	US_tissue_wavelet3-LH_firstorder_Uniformity
MGcc_tissue_wavelet2-HL_ glcm_ClusterTendency	US_tissue_wavelet2-LH_firstorder_Kurtosis	MGmlo_tissue_original_shape2D_ Sphericity	US_tissue_exponential_glszm_ ZonePercentage
MGcc_tissue_gradient_firstorder_Skewness	US_tissue_wavelet-HH_glszm_ SmallAreaLowGrayLevelEmphasis	MGcc_tissue_gradient_firstorder_Skewness	MGmlo_tissue_original_shape2D_ Sphericity
MGmlo_tissue_wavelet3-LL_gldm_DependenceVariance	US_tissue_wavelet3-HH_glrlm_RunLengthNonUniformityNormalized	US_tissue_wavelet2-LH_glrlm_RunLengthNonUniformityNormalized	MGcc_tissue_gradient_firstorder_ Skewness
MGmlo_tissue_exponential_ glcm_Correlation	US_tissue_logarithm_gldm_ SmallDependenceLowGrayLevelEmphasis	MGmlo_tissue_wavelet3-HH_ glcm_Correlation	US_tissue_wavelet2-LH_glrlm_ RunLengthNonUniformityNormalized
MGmlo_tissue_wavelet2- HL_firstorder_Skewness	US_tissue_wavelet2-HH_glszm_SmallAreaLowGrayLevelEmphasis	US_tissue_original_shape2D_ Sphericity	MGmlo_tissue_wavelet3-HH_ glcm_Correlation
MGcc_tissue_wavelet-LH_ glcm_MCC	US_tissue_wavelet-HH_glcm_ MaximumProbability	US_tissue_wavelet3-HH_glrlm_RunLengthNonUniformityNormalized	MGcc_tissue_exponential_glcm_ DifferenceAverage
MGmlo_tissue_exponential_ glcm_DifferenceVariance	US_tissue_gradient_glcm_Imc2	MGmlo_tissue_exponential_ firstorder_Kurtosis	MGmlo_tissue_exponential_ firstorder_Kurtosis
MGcc_tissue_exponential_ ngtdm_Strength	US_tissue_square_firstorder_Variance	MGcc_tissue_exponential_glcm_ DifferenceAverage	US_tissue_wavelet3-HH_glrlm_RunLengthNonUniformityNormalized
MGcc_tissue_wavelet-LH_ firstorder_Kurtosis	US_tissue_wavelet2-LH_gldm_LargeDependenceLowGrayLevelEmphasis	US_tissue_wavelet-HL_ngtdm_ Strength	US_tissue_original_shape2D_ Sphericity
MGcc_tissue_wavelet3-LH_gldm_DependenceVariance	US_tissue_squareroot_firstorder_ Minimum	US_tissue_exponential_glcm_Idmn	US_tissue_wavelet-HL_ngtdm_ Strength
MGcc_tissue_exponential_glrlm_GrayLevelNonUniformity	US_tissue_logarithm_glcm_Idn	US_tissue_gradient_glszm_ GrayLevelNonUniformityNormalized	US_tissue_exponential_glcm_Idmn
MGmlo_tissue_wavelet-HL_ firstorder_Minimum		US_tissue_logarithm_gldm_ SmallDependenceLowGrayLevelEmphasis	Vascularity grade
MGmlo_tissue_wavelet3- HH_glcm_MCC		US_tissue_wavelet3-LH_glrlm_ RunVariance	US_tissue_gradient_glszm_ GrayLevelNonUniformityNormalized
		US_tissue_wavelet2-HH_glszm_ SmallAreaLowGrayLevelEmphasis	US_tissue_logarithm_gldm_ SmallDependenceLowGrayLevelEmphasis
		US_tissue_wavelet2-HH_glrlm_ RunVariance	MGcc_tissue_wavelet3-HH_ glcm_DifferenceVariance
		MGcc_tissue_wavelet3-HH_glcm_DifferenceVariance	US_tissue_wavelet3-LH_glrlm_ RunVariance
		US_tissue_square_firstorder_Variance	US_tissue_square_firstorder_ Variance
		MGmlo_tissue_wavelet3-LL_ gldm_DependenceVariance	US_tissue_wavelet-HH_glszm_SmallAreaLowGrayLevelEmphasis
		US_tissue_wavelet-HH_glcm_ MaximumProbability	MGmlo_tissue_wavelet3-LL_ gldm_DependenceVariance

MG, mammography; GSUS, gray-scale ultrasound; Rad-MG-GSUS model, the model based on radiomics features from MG and GSUS images; Rad-MG model, the model based on radiomics features from MG images; Rad-GSUS model, the model based on radiomics features from GSUS images; combined model, the model based on clinical features, MG and GSUS radiomics features.

### Comparison of performance between models

No significant differences were noted between the Rad-GSUS model and the Rad-MG model in the training cohort (AUCs 0.936 vs. 0.974; *p*=0.166) and the test cohort (AUCs 0.876 vs. 0.885; *p*=0.926). The Rad-MG-GSUS model performed better than the Rad-GSUS model in the training cohort (AUCs 0.991 vs. 0.936; *p*=0.021). Although the Rad-MG-GSUS model showed improved efficacy over the Rad-MG model in both the training cohort (AUCs 0.991 vs. 0.974; *p*=0.174) and the test cohort (AUCs 0.949 vs. 0.885; *p*=0.322), as well as over the Rad-GSUS model in the test cohort (AUCs 0.949 vs. 0.876; *p*=0.093), the differences were not statistically significant.

**Figure 6:**
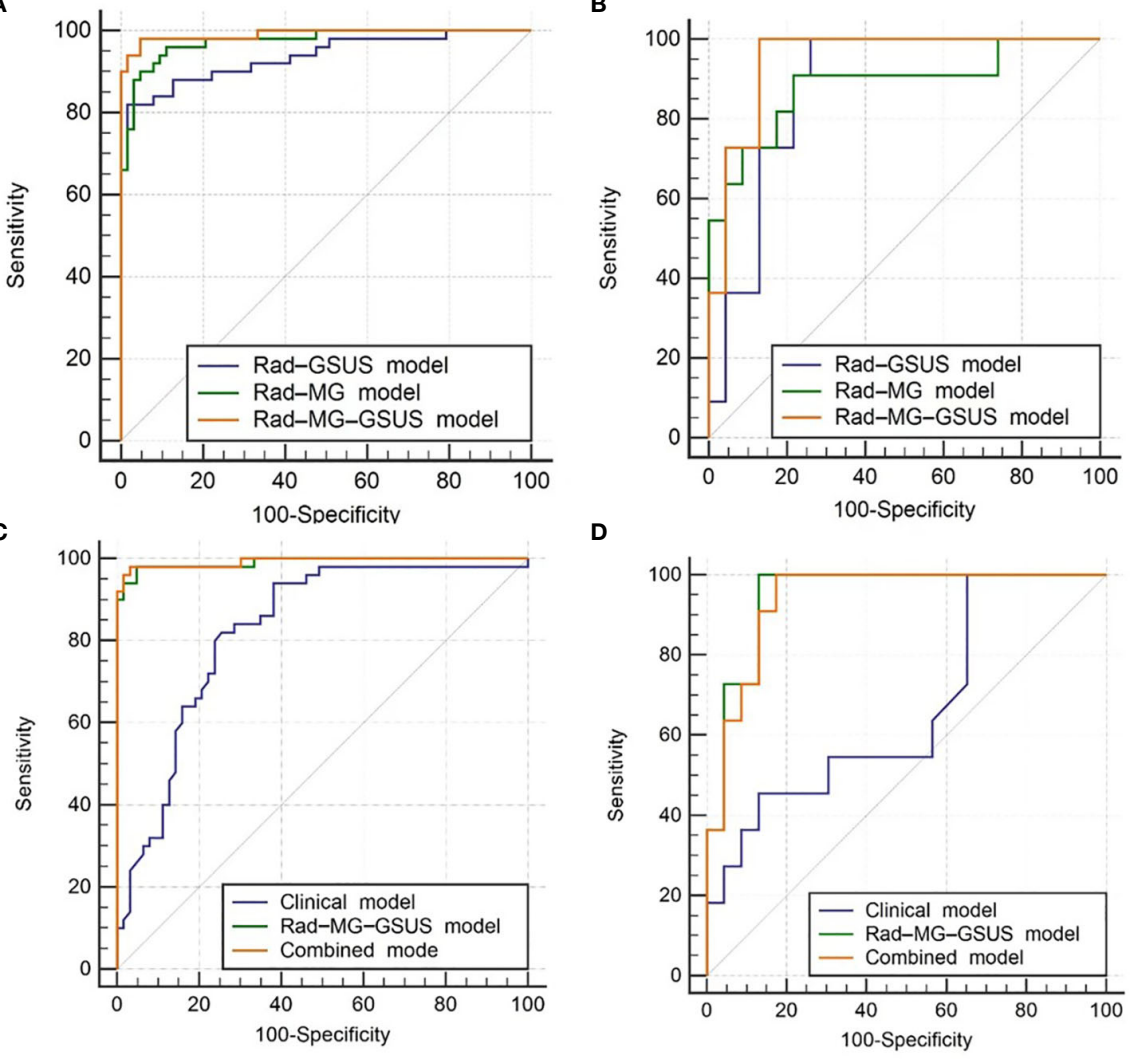
Receiver Operating Characteristic (ROC) Curves. **(A)** ROC curves for three radiomics models in the training cohort; **(B)** ROC curves for three radiomics models in the test cohort; **(C)** ROC curves for the clinical model, Rad-MG-GSUS model, and combined model in the training cohort; **(D)** ROC curves for the clinical model, Rad-MG-GSUS model, and combined model in the test cohort. MG, mammography; GSUS, gray-scale ultrasound. Rad-MG-GSUS model: the model based on radiomics features from MG and GSUS images; Rad-MG model: the model based on radiomics features from MG images; Rad-GSUS model: the model based on radiomics features from GSUS images; combined model: the model based on clinical features, MG and GSUS radiomics features.

**Figure 7:**
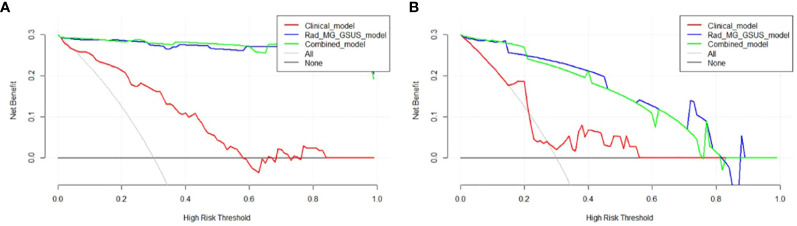
Decision Curve Analysis (DCA) of Three Models. **(A)** DCA curves for three models in the training cohort; **(B)** DCA curves for three models in the test cohort. The y-axis measures the net benefit, calculated as the difference between expected benefit and harm associated with each proposed model. If the threshold probability exceeds 2%, using the models to predict breast adenosis provides a higher net benefit than scenarios without a prediction model.

The combined model and the Rad-MG-GSUS model showed significantly higher AUCs than the clinical model in both the training cohort (AUCs 0.993 vs. 0.826, *p*<0.001; AUCs 0.991 vs. 0.826, *p*<0.001, respectively) and the test cohort (AUCs 0.941 vs. 0.664, *p*=0.014; AUCs 0.949 vs. 0.664, *p*=0.011, respectively). No significant differences were noted between the combined model and the Rad-MG-GSUS model in the training cohort (AUCs 0.993 vs. 0.991; *p*=0.212) and the test cohort (AUCs 0.941 vs. 0.949; *p*=0.299). The performance of the five models is summarized in **Table 3**. The comparison of predictive models is summarized in **Table 4**, and the ROC curves of the models are depicted in **Figure 6A-D**. DCA shows comparable clinical benefits for the Rad-MG-GSUS model and the combined models over a large range of threshold probabilities in **Figure 7**.

**Table 3:** Diagnostic performance of the five models.

Different models	AUC (95%CI)	Training cohort		Accuracy	AUC(95%CI)	test cohort		Accuracy
		Sensitivity	Specificity			Sensitivity	Specificity	
Rad-MG model	0.974(-0.949, -0.999)	0.920	0.921	0.920	0.885(-0.746, -1.00)	0.909	0.565	0.676
Rad-GSUS model	0.936(-0.888, -0.983)	0.820	0.921	0.876	0.876(-0.761, -0.993)	1.000	0.652	0.765
Rad-MG-GSUS model	0.991(-0.976, -1.00)	0.940	0.952	0.947	0.949(-0.879, -1.00)	1.000	0.869	0.912
clinical model	0.826(-0.747, -0.903)	0.820	0.730	0.770	0.664(-0.459, -0.868)	0.545	0.652	0.618
Combined model	0.993(-0.980, -1.00)	0.960	0.968	0.965	0.941(-0.866, -1)	1.000	0.826	0.882

MG: mammography; GSUS: gray-scale ultrasound; Rad-MG-GSUS model: the model based on radiomics features from MG and GSUS images; Rad-MG model: the model based on radiomics features from MG images; Rad-GSUS model: the model based on radiomics features from GSUS images; combined model: the model based on clinical features, MG and GSUS radiomics features.

**Table 4:** Performance comparison of predictive models in the training cohort and the test cohort.

Models	training cohort			Models	test cohort		
	AUC	*Z* statistic	*p*-value		AUC	*Z* statistic	*p*-value
Rad-MG vs Rad-GSUS	0.974 vs 0.936	1.386	0.166	Rad-MG vs Rad-GSUS	0.885 vs 0.876	0.093	0.926
Rad-MG vs Rad-MG-GSUS	0.974 vs 0.991	1.36	0.174	Rad-MG vs Rad-MG-GSUS	0.885 vs 0.949	0.99	0.322
Rad-GSUS vs Rad-MG-GSUS	0.936 vs 0.991	2.317	0.021^*^	Rad-GSUS vs Rad-MG-GSUS	0.876 vs 0.949	1.68	0.093
Clinical vs Combined	0.826 vs 0.993	4.138	<0.001^*^	Clinical vs Combined	0.664 vs 0.941	2.461	0.014^*^
Rad-MG-GSUS vs Combined	0.991 vs 0.993	1.248	0.212	Rad-MG-GSUS vs Combined	0.949 vs 0.941	1.038	0.299
Rad-MG-GSUS vs Clinical	0.991 vs 0.826	4.057	<0.001^*^	Rad-MG-GSUS vs Clinical	0.949 vs 0.664	2.551	0.011^*^

*P value < 0.05, with statistical difference.

MG, mammography; GSUS, gray-scale ultrasound; Rad-MG-GSUS model, the model based on radiomics features from MG and GSUS images; Rad-MG model, the model based on radiomics features from MG images; Rad-GSUS model, the model based on radiomics features from GSUS images; combined model, the model based on clinical features, MG and GSUS radiomics features; AUC, areas under the receiver operator characteristics curve.

## Discussion

Accurate qualitative diagnosis of breast adenosis using GSUS or MG remains challenging for radiologists (25). This study utilized GSUS-based and MG-based radiomics features to differentiate breast adenosis from IDC. The interpretability of the model was an important factor in our consideration. LR model provides easy-to-interpret coefficients that can reflect the importance of each feature to decision making, and LR is the most commonly used classification algorithm in clinical research. The results demonstrated that LR models incorporating GSUS-based and MG-based radiomics features could effectively distinguish breast adenosis from IDC. Notably, the Rad-MG-GSUS model outperformed the Rad-GSUS and Rad-MG models. Additionally, a combined model integrating clinical parameters and radiomics features from MG and GSUS images was developed and validated. Compared to the clinical model, both the combined model and the Rad-MG-GSUS model exhibited superior performance. The predictive performance differences between the Rad-MG-GSUS model and the combined model in both the training and test cohorts were not significant. DCA further indicated increased net clinical benefits for these models compared to no prediction models, offering potential assistance in devising improved treatment plans for patients.

Univariate analysis revealed that breast adenosis was more prevalent in younger women without menopausal symptoms, less common with II~III vascularity grade on ultrasound, and associated with the absence of a family history and fine linear/fine-linear branching on mammography. These findings align with previous studies (8, 9, 26–28). Notably, family history and vascularity grade features were retained during feature selection in the combined model, suggesting a significant correlation with breast adenosis. A family history of breast cancer is a significant risk factor, accounting for approximately 5-10% of breast cancer cases (26). Tumor angiogenesis, an increase in blood flow and the presence of irregular or penetrating blood vessels are responsible for the development and progression of cancer (29). However, in the test cohort, the clinical model demonstrated an AUC of only 0.664, potentially influenced by the small number of included patients. Further investigation with larger and more relevant studies may be necessary to validate these findings.

Recently, due to the standardization of radiomics methodologies and the development of tools, as well as the widespread acceptance of the idea, radiomics has become extensively utilized in all parts of tumor diagnostics (30, 31). Previously, radiomics research often utilized single-modality or single-sequence images. However, the effectiveness of radiomics that based on single-modality medical images, which only captures a portion of tumor information due to its imaging principles, has been unavoidably degraded (14–16). Radiomics based on multimodal images extracts various aspects of information from each modal image and then combine them for model development, gaining increasing interest (16, 17). Tan et al. (32) evaluated the individual and combined efficacy of artificial intelligence (AI) detection systems for digital mammography and automated 3D breast ultrasound in the identification of breast cancer in women with dense breasts. The study revealed that the AI systems performed significantly better when operating in a multi-modal setting compared to when each system operated individually in a single-modal setting (AUC-AI -Multimodal =0.865; AUC-AI-DM=0.832, *p*=0.026; AUC -AI- ABUS=0.841, *p*=0.041). Zheng et al. (7) conducted a study to assess the clinical usefulness of a radiomics model that utilizes GSUS and contrast-enhanced ultrasound (CEUS) images for distinguishing between inflammatory mass stage periductal mastitis/duct ectasia (IMSPDM/DE) and IDC. The study discovered that the GSUS combined with CEUS radiomics signature outperformed the other two radiomics signatures. Similar to our findings, the combination of the two modalities demonstrates exceptional discriminatory ability (AUC of 0.941 in the test cohort).

Our finding suggest that both MG-based and GSUS-based radiomics features effectively distinguish breast diseases and IDC, yielding AUCs of 0.885 and 0.876 in the respective test cohorts. Additionally, a radiomics model utilizing ultrasound, as devised by Huang et al. (33), exhibits robust diagnostic performance for sclerosing adenosis and invasive ductal carcinoma, with AUCs of 0.886 and 0.779 in the validation and independent validation cohorts. A prior investigation demonstrates that employing radiomics data from both CC and MLO positions outperforms using CC or MLO positions alone in classification (34). Consequently, we utilized radiomics data from both CC and MLO positions to differentiate breast adenosis and IDC. The results indicate that the Rad-MG model achieves commendable classification performance. The radiomics models included shape radiomics features from both MG and GSUS, as two-dimensional images from these modalities provide limited tumor morphological information. By including shape radiomics features from MG and GSUS with statistically significant differences, we aim to offer a more comprehensive view of tumor morphology from different angles. To reduce feature redundancy, we applied the mRMR method, ensuring a more effective feature selection process.

Our research shows that the clinical model exhibited moderate efficacy in distinguishing between breast adenosis and IDC. A significant enhancement in efficacy was observed when combining clinical data with radiomics features to form a composite model. This suggests that radiomics have the capacity to enhance the objectivity of image representation by emphasizing graphical features that are not visible to the human eye. In the test cohort, the AUC value of the combined model was estimated to be 0.941, somewhat lower than that of the Rad-MG-GSUS model (AUC value: 0.949), however, the difference between the two values was not statistically significant. We believe this might be due to the excellent diagnostic efficiency of the Rad-MG-GSUS model, resulting in the addition of clinical information not enhancing the diagnostic efficiency of the combined model, or due to the sample size or the characteristics of patients within the cohort.

Our study has several limitations. Firstly, being a retrospective single-center study introduces the possibility of selection bias. Future validation with larger sample sizes and external test cohorts is essential. Secondly, the study exclusively considered GSUS and MG features, while MRI is another crucial method for detecting breast disease. Exploring the potential comprehensive information offered by the combination of all three modalities warrants investigation in subsequent studies. Thirdly, different ultrasound equipment and scanning parameters may impact the generality of the results. Finally, to ensure precise correspondence between the lesions analyzed in the images and those obtained from surgical or biopsy specimens, we did not include cases with multifocal lesions in our study. Therefore, if multifocal lesions are of the same pathological type, would there be significant differences in the radiomic features between the lesions? A dedicated study is required to verify this.

## Conclusions

In conclusion, GSUS and MG radiomic features demonstrate outstanding performance in distinguishing between breast adenosis and IDC. The amalgamation of radiomic features from both modalities, along with clinical features, enhances identification efficacy. This could serve as a valuable reference in the clinical decision-making process.

## Data availability statement

The original contributions presented in the study are included in the article/supplementary material. Further inquiries can be directed to the corresponding authors.

## Ethics statement

The studies involving humans were approved by the institutional ethics committee of the First Affiliated Hospital of Soochow University. The studies were conducted in accordance with the local legislation and institutional requirements. The ethics committee/institutional review board waived the requirement of written informed consent for participation from the participants or the participants’ legal guardians/next of kin because This retrospective study received approval from our institution’s independent ethics committee, and the need for informed consent was waived.

## Author contributions

WL: Writing – original draft, Data curation, Conceptualization. YS: Writing – original draft, Data curation. XQ: Writing – review & editing, Validation, Formal Analysis, Conceptualization. HZ: Writing – original draft, Investigation, Formal Analysis. LS: Writing – review & editing, Data curation. YD: Writing – original draft, Visualization, Software. FD: Writing – review & editing, Methodology, Data curation. YL: Writing – original draft, Supervision, Methodology, Funding acquisition, Conceptualization. LZ: Writing – original draft, Data curation, Investigation.
